# The role of hepatic sinusoidal microenvironment in NASH: pathogenesis, animal models, and therapeutic prospects

**DOI:** 10.3389/fphar.2025.1467950

**Published:** 2025-04-28

**Authors:** Wanying Tan, Jiangting Deng, Lingjun Qi, Zhenghuai Tan

**Affiliations:** ^1^ Center of Infectious Diseases, West China Hospital of Sichuan University, Chengdu, Sichuan, China; ^2^ Sichuan Academy of Chinese Medicine Sciences, Sichuan Provincial Key Laboratory of Quality and Innovation Research of Chinese Materia Medica, Chengdu, Sichuan, China; ^3^ School of Pharmacy, Chengdu University of Traditional Chinese Medicine, Chengdu, Sichuan, China; ^4^ Affiliated Sichuan Gem Flower Hospital of North Sichuan Medical College, Chengdu, Sichuan, China

**Keywords:** nonalcoholic steatohepatitis, pathogenesis, animal model, drug development, hepatic sinusoidal microenvironment

## Abstract

The incidence of nonalcoholic steatohepatitis (NASH) is increasing annually, posing a significant threat to human health. NASH is typified by hepatic steatosis, inflammation, and hepatocellular injury, frequently culminating in fibrosis and cirrhosis. Yet, the precise pathogenesis of NASH remains to be fully elucidated. The hepatic sinusoid, which serves as the fundamental structural and functional unit of the liver, is intricately composed of endothelial cells, Kupffer cells, and hepatic stellate cells. Consequently, the homeostasis of the hepatic sinusoidal microenvironment may exert a pivotal influence on the progression and prognosis of NASH. However, the limitations of current NASH animal models have significantly impeded advancements in understanding the disease’s pathogenesis and the development of effective therapeutic interventions. In light of these challenges, this review endeavors to delve deeper into the critical role of hepatic sinusoidal microenvironment homeostasis in the pathogenesis of NASH, critically analyze the commonly employed animal models, and comprehensively summarize the most recent and promising developments in drug research and development. It is anticipated that these efforts will collectively expedite the advancement of the field of NASH research and therapeutic innovation.

## 1 Introduction

Nonalcoholic fatty liver disease (NAFLD) should be replaced by metabolic dysfunction-associated steatotic liver disease (MASLD), which relies on evidence of fatty liver in conjunction with one of the following conditions (Eslam et al., 2020): overweight or obesity (Rinella et al., 2023), metabolic dysregulation characterized by at least two risk factors (Anstee et al., 2019), or the presence of type 2 diabetes mellitus (T2DM) (Eslam et al., 2020). In 2023, three large multinational liver associations subsequently recommended that the term MASLD should supplant the designation NAFLD (Rinella et al., 2023). However, the results from patients with NASH, NASH animal models, and metabolism-regulating drugs for NASH have not yielded successful outcomes, implying that metabolic disorders are crucial in the onset and progression of NASH but are not the sole determining factor. According to the literature referenced in this article, the terms NAFLD or NASH will continue to be utilized in the subsequent relevant content descriptions to preserve the original intent of the author.

NAFLD is currently the most common cause of chronic liver disease worldwide, with an incidence rate ranging from 25% to 30% (Anstee et al., 2019). NASH is an advanced form of NAFLD, with a global prevalence of 5.27%, characterized by the accumulation of toxic lipids in the liver, leading to inflammation and hepatocyte injury (Younossi et al., 2023). NASH can further progress to end-stage liver diseases such as cirrhosis and hepatocellular carcinoma (Kanwal et al., 2018), but there are currently no ideal effective drugs for prevention and treatment. The main reasons are related to the unclear pathogenesis of the disease and the lack of ideal animal models.

The hepatic sinusoid is an important structural unit in the maintenance of the homeostasis of the microenvironment in the liver, which is the main place for material exchange between blood and the Disse space (Rojkind et al., 1988). The hepatic sinusoidal niche, comprising Kupffer cells (KCs), liver sinusoidal endothelial cells (LSECs), hepatic stellate cells (HSCs), and hepatocytes, is central to this process. LSECs form the hepatic sinusoidal endothelium, KCs reside within the sinusoidal lumen and respond to external stimuli, while HSCs are located in the space of Disse and interact closely with LSECs to regulate blood flow, cellular trans-sinusoidal migration, and lipoprotein exchange. The liver can experience pathological processes such as inflammation, fibrosis, microvascular thrombosis, and portal hypertension due to the instability of the hepatic sinus microenvironment. Although the mechanism of simple fatty liver deteriorating to NASH could be explained by the two-hit hypothesis, the multiple parallel hits hypothesis, or the revised multiple parallel hits hypothesis (Tilg and Moschen, 2010), the pathogenesis of NASH remains incompletely understood, with the progression from simple steatosis to NASH not yet fully elucidated. The homeostasis of the hepatic sinusoidal niche is also maintained through the interplay of the sympathetic and parasympathetic nervous systems. Despite their critical roles in NASH development, these mechanisms are underreported. Thus, investigating the hepatic sinusoidal niche and its neural regulation may provide valuable insights into NASH pathogenesis. This review aims to further explore the important role of hepatic sinus microenvironment homeostasis in the pathogenesis of NASH, analyze commonly used animal models, and summarize relevant promising drug research and development progress.

## 2 Maintaining the homeostasis of the hepatic sinusoidal microenvironment in NASH

The primary constituents of the hepatic sinus microenvironment are the KCs, LSECs, HSCs, and hepatocytes. LSECs form the microvascular wall of the hepatic sinusoid, while KCs are located in the sinusoidal cavity and can respond to external stimuli. HSCs are located in the Disse space and are tightly connected to LSECs within the Disse space to regulate blood flow, cell migration across sinuses, and the exchange of lipoprotein particles (Brunt et al., 2014).

### 2.1 Macrophages

Macrophages in the liver are categorized into two phenotypes: KCs and monocyte-derived macrophages (MoMFs). Under normal conditions, KCs are usually tolerant; they regulate cholesterol homeostasis by producing cholesterol ester transfer proteins, mediating antimicrobial defense, and promoting immune tolerance (Wang Y. et al., 2015; Helmy et al., 2006; Zeng et al., 2016). NOD-like receptor thermal protein domain-associated protein 3 (NLRP3)is a multi-protein scaffold mainly expressed in KCs. Free fatty acids (FAs) such as palmitates coordinate with Toll-like receptor 2 and Toll-like receptor 9 ligands to activate KCs through NLRP3 inflammasome. Once activated, NLRP3 will expand inflammatory body-driven fibrosis and activate NLRP3 inflammasome launching or aggravating liver inflammation by activating hepatic caspase-1 and producing excessive interleukin-1β (IL-1β) and reactive oxygen species (ROS) (Gaul et al., 2021; Yang et al., 2021).

During inflammatory situations, a large number of monocytes from the bone marrow are recruited, leading to a surge in liver MoMFs (Scott et al., 2016). After entering the liver, MoMFs will differentiate into two subsets: Ly6C^high^ and Ly6C^low^. The Ly6C^high^ type can promote liver inflammation and fibrosis, while the Ly6C^low^ type has an anti-fibrosis effect (Hashimoto et al., 2013). During NASH and liver cirrhosis, the number of KCs in the liver decreases, and MoMFs will gradually recruit and infiltrate the liver to maintain the number of macrophage pools in the liver (Krenkel et al., 2018).

NAFLD progresses to the fibro-inflammatory stage due to the activation of these macrophages (Alabdulaali et al., 2023). Tumor necrosis factor-α (TNF-α), transforming growth factor-β (TGF-β), platelet-derived growth factor, IL-1β, and CC-chemokine ligand (CCL) 2 secreted from the activated hepatic macrophages promote the transformation of HSCs into activated myofibroblasts (Krenkel et al., 2018; Marra and Tacke, 2014; Pradere et al., 2013). Macrophage c-mer tyrosine kinase, which is on the surface of liver macrophages, increases the survival of myofibroblasts and accelerates liver fibrosis *via* the TGF-β signal path (Pradere et al., 2013; Cai et al., 2020).

### 2.2 LSECs

LSECs are endothelial cells with a high degree of differentiation, serving as the vascular walls of the liver microcirculation system (Marrone et al., 2016). They have unique characteristics, including fenestration without a basement membrane, and demonstrate properties of reducing inflammation and preventing fibrosis (Wisse et al., 1985). LSECs have many functions, such as transferring lipoproteins, small chylomicron residues, and other large molecules from the sinus vascular side to the Disse space through fenestration, thereby being absorbed by liver cells (Hilmer et al., 2005). They also have a barrier effect on certain pathogens, such as clearing viruses, bacteriophages, or lipopolysaccharide (Wang and Liu, 2021). Most importantly, LSECs mediate hepatic vascular function by inhibiting the activation of KCs and HSCs, thereby inhibiting liver inflammation (Du and Wang, 2022). In addition to regulating the expression of endothelial nitric oxide synthase (eNOS) and maintaining liver homeostasis, LSECs can activate the transcription factor Kruppel-like factor 2 and synthesize nitric oxide (NO) (Gracia-Sancho et al., 2021).

During the initial phase of NAFLD, excessive lipids, carbohydrates, and gut microbiota may cause capillarization of LSECs (Cogger et al., 2016). LSECs undergo a swift capillary transformation, marked by the vanishing of fenestrae, the formation of the basement membrane, and the emergence of distinctive markers (Cogger et al., 2016). As NASH evolves, LSECs show a pro-inflammatory phenotype, which is characterized by enhancing the expression of vascular adhesion protein 1, platelet endothelial cell adhesion molecule 1, e-selectin, intercellular adhesion molecule 1, cyclooxygenase 2, IL-6, nicotinamide adenine dinucleotide phosphate-oxidase 2, and TNF-α (Kus et al., 2019). Furthermore, in response to the lipotoxicity reaction induced by palmitic acid or oxidized low-density lipoprotein, the LSECs produce ROS and aggravate liver inflammation (Hammoutene et al., 2020; Matsumoto et al., 2018). The soluble circulating form of adhesion molecules released by LSECs with pro-inflammatory phenotype may be a potential non-invasive biomarker in human NASH.

In addition, the dysfunctional LSECs decrease the eNOS activity and NO production, which stimulates the synthesis of citrate and lipids and increase the content of triglyceride (TG) in the liver (Tateya et al., 2011). The decreases in eNOS activity on capillarized LSECs lead to a decrease in NO production and bioavailability, which limits vascular dilation and reduces liver blood flow (Flammer et al., 2012).

### 2.3 HSCs

In their physiological state, HSCs have five functions: promoting liver development and regeneration, participating in the metabolism and storage of vitamin A, maintaining extracellular matrix (ECM) homeostasis, secreting cytokines, and participating in drug metabolism and detoxification to protect hepatocytes from injury (Puche et al., 2013). HSCs secrete laminin, types III and IV collagen, various matrix metalloproteinases, and tissue inhibitors of metalloproteinases, and their interactions maintain liver matrix renewal, homeostasis, and healthy liver architecture.

HSCs are the dominant cells in liver fibrosis. At the beginning, the resting HSCs are activated to become the myofibroblast phenotype, which is characterized by significant upregulation of α-smooth muscle actin, desmin, and type I collagen (Lepreux and Desmoulière, 2015). As the proliferation and survival of HSCs intensify, the enhancement of ECM synthesis occurs in order to react to chemical attractants, stimulate inflammatory response, control immune response, and acquire contractility (Garbuzenko, 2022). Myofibroblasts are the primary producers of collagen and other ECM proteins (such as type I and III collagen, as well as other proteins that make up pathological fibrous tissue) and, therefore, play a dominant role in scar formation during liver fibrosis (Higashi et al., 2017). ECM deposition increases its cross-linking, making it more difficult to degrade, and further development can lead to scar deposition and liver function damage (Kumar et al., 2021).

Early activation of HSCs is susceptible to cytokines and extrahepatic stimuli. Injured hepatocytes initially produce IL-33, which activates and aggregates innate lymphoid cells 2 through ST2-dependent signals, and produces IL-13, which triggers HSCs activation and trans-differentiation in a manner dependent on IL-4Rα and STAT6 transcription factors (McHedlidze et al., 2013). The activation of non-infectious inflammation and HSCs is initiated and sustained by the release of inflammatory mediators, including TNF-α, IL-1β, IL-6, ROS, hedgehog ligands, and nucleotides, from dying or decaying epithelial cells and their phagocytic leukocytes (Heymann and Tacke, 2016). In addition to activating HSCs, ROS also stimulate signaling pathways and transcription factors, increasing the expression of fibrogenic genes in HSCs, including collagen type I alpha 1, collagen type I alpha 2, monocyte chemoattractant protein 1, and tissue inhibitors of metalloproteinases 1 (Liu et al., 2013).

During the progression of NASH, hepatocyte apoptosis and the immune-regulatory factors produced by macrophages recruited through efferocytosis play a pivotal role in the activation of HSCs and the subsequent development of fibrosis (Dewidar et al., 2019). Activated HSCs also further stimulate other resting HSCs through autocrine TGF-β, and TGF-β can be activated by sediment present in the ECM, which leads to its expression and release by various cell types, ultimately contributing to the formation of persistent fibers within the feedforward loop.

### 2.4 Crosstalk between cells in the liver-sinus microenvironment

**FIGURE 1:**
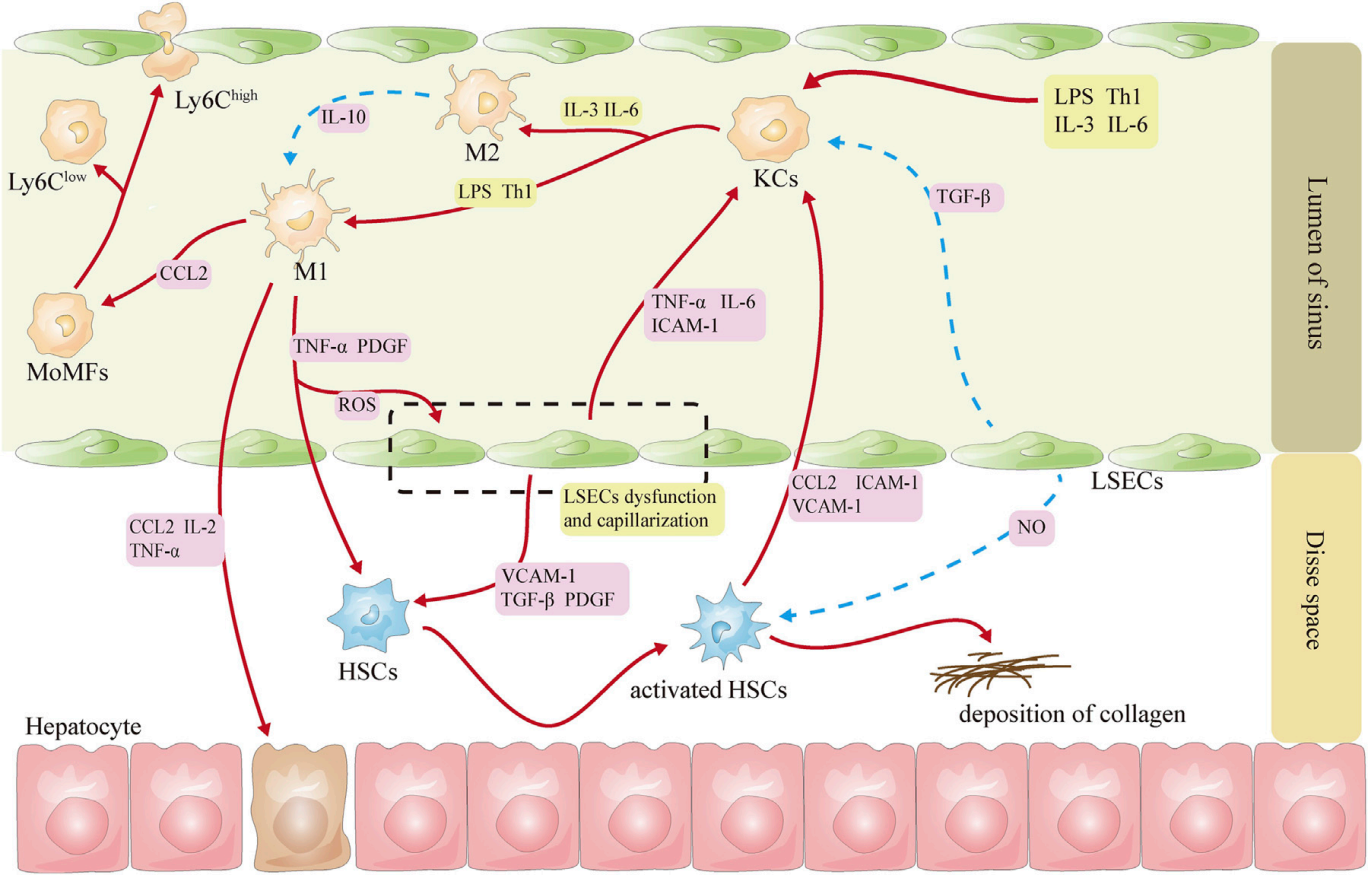
Schematic diagram of the interactions among KCs, LSECs, and HSCs. Note (Eslam et al., 2020): Red lines represent activation (Rinella et al., 2023), and blue lines represent inhibition (Anstee et al., 2019). CCL2, CC-chemokine ligand 2; HSCs, hepatic stellate cells; ICAM-1, intercellular adhesion molecule 1; IL-2: interleukin-2; IL-3, interleukin-3; IL-6: interleukin-6; KCs, Kupffer cells; LPS, lipopolysaccharide; LSECs, liver sinusoidal endothelial cells; NO, nitric oxide; PDGF, platelet-derived growth factor; ROS: reactive oxygen species; Th1, helper T cells 1; TNF-α, tumor necrosis factor-α; VCAM-1, vascular cell adhesion molecule 1; TGF-β, transforming growth factor β.

Hepatic sinusoids are the best environment for cellular function and communication in the liver, especially KCs, LSECs, and HSCs, and the interaction among them is essential for the maintenance of hepatic homeostasis (Figure 1).

Under physiological conditions, Notch ligands delta-like 4 and TGF-β derived from LSECs can inhibit the activation of KCs, thereby maintaining KCs in a nonpolarized state (Sakai et al., 2019). After LSECs capillarization, KCs are activated, which in turn increases the expression of chemokine CCL2 and the recruitment of MoMFs in the liver (Schwabe et al., 2020). Activated KCs increase the expression of adhesion molecules on LSECs by releasing damage-related molecular patterns, cytokines, and inflammatory factors and participate in the capillary action of LSECs, leading to subsequent recruitment of white blood cells (Coulon et al., 2011; Ford et al., 2015; Shetty et al., 2018). LSECs can protect the quiescent state of HSCs by secreting NO (García-Lezana et al., 2018). During the stage of NASH, LSECs may activate HSCs *via* releasing TGF-β, platelet-derived growth factor, hedgehog ligand, and vascular cell adhesion molecule 1 (Guo Q. et al., 2022). Capillarized LSECs may impair the blood oxygen exchange and lead to hypoxia in the microenvironment, which will further induce the rapid activation of HSCs and the expression of hypoxia-inducible factor 1α (Wan et al., 2019). Dedifferentiated LSECs also affect the activation and movement of HSCs through the release of exosomes containing abundant sphingosine kinase 1 (Wang R. et al., 2015). In turn, activated HSCs can also alter the expression of the LSECs gene by releasing exosomes rich in Hedgehog (Witek et al., 2009).

In the liver, HSCs can be activated directly by both KCs and Ly6C^high^ MoMFs through the secretion of various cytokines and chemokines (Krenkel et al., 2018; Marra and Tacke, 2014; Pradere et al., 2013). The activated HSCs secrete CCL2 and M-CSF, intercellular adhesion molecule 1, vascular cell adhesion molecule 1, and E-selectin, inducing the activation and migration of KCs (Gluchowski et al., 2017). The Ly6C^low^-type MoMFs may promote apoptosis of HSCs (Li et al., 2020), although the mechanism is not currently understood.

### 2.5 Action of the nervous system on hepatic sinusoids

In addition to interactions between cells of the hepatic sinusoids through direct contact or cytokine delivery, hepatic sinusoidal microenvironmental homeostasis may be maintained through interactions between sympathetic and parasympathetic nerves. Although hepatic progenitor cells are activated and differentiate into hepatocytes after severe liver injury, their proliferation can be enhanced by inhibiting the sympathetic nervous system, resulting in amelioration of hepatic injury (Xue et al., 2024). In contrast, vagotomy impairs liver regeneration (Lam et al., 2008).

It was found that there is autonomic innervation in hepatic sinus tissue, in which the sympathetic nerve mainly releases monoamine neurotransmitters, while the parasympathetic nerve mainly releases choline neurotransmitters. The acetylcholine (ACh) released from the parasympathetic nerve can act on LSECs and cause hepatic sinus dilation, while norepinephrine (NE) from sympathetic may induce hepatic sinus contraction (Mizuno and Ueno, 2016; Ueno et al., 2004).

By enhancing the phagocytic activity of KCs, the vagus nerve promotes the clearance of microorganisms by the liver and facilitates the development of pro-regenerative and anti-inflammatory characteristics (Fonseca et al., 2019). Moreover, the vagus nerves are essential in facilitating FAs synthesis-induced hepatocyte apoptosis through their modulation of KCs and attenuation of ROS production (Bonaz et al., 2019). The α7 nicotinic acetylcholine receptors (a7nAChR) are expressed on KCs and hepatocytes. ACh decreases the release of inflammatory cytokines by activating a7nAChR on KCs. In NASH, a7nAChR agonists have been shown to inhibit the inflammatory response in KCs. Conversely, the lack of a7nAChR in KCs exacerbates NASH-related inflammatory reactions and abnormal lipid metabolism, as evidenced by markedly heightened levels of TNF-a, IL-12, and monocyte chemoattractant protein 1 in the liver (Nishio et al., 2017). KCs and hepatocytes, respectively, enhance the production of TNF‐α and IL‐6 in response to NE. The sympathetic nervous system can promote hepatic inflammatory responses by accelerating the secretion of IL-6 and TGF-β from KCs through stimulation of α1-adrenergic receptors, as well as by releasing neurotransmitters to promote KCs activation (Huan et al., 2017).

Because of the long-branched cell processes and the special anatomical position, HSCs can contact nerve endings in time. A number of factors can cause HSCs to contract, including endothelin 1, angiotensin II, NE, thromboxane A2, platelet-activating factor, and thrombin. Meanwhile, prostaglandin E2, CO, sulfur dioxide, hydrogen sulfide, O_2_, ACh vasoactive intestinal peptide, and adrenomedullin have the ability to relax HSCs. ACh generated by the hepatic vagus nerve stimulates hepatic fibrosis through the promotion of HSCs proliferation and upregulation of collagen gene expression (Oben et al., 2003). In galactosamine and lipopolysaccharide (LPS)-induced liver damage, vagotomy, and atropine inhibited HSCs activation and proliferation, while stimulation of the vagus nerve increased HSCs proliferation (Bockx et al., 2010). HSCs possess adrenoceptors, and the growth of HSCs is accelerated by sympathetic stimulation, a feature involved in the progression of hepatic fibrosis (Medina Pizaño et al., 2023). The proliferation of human HSCs was induced by exogenous NE/adrenaline and neuropeptide Y through the activation of P38 Mitogen-activated protein kinase (MAPK), phosphatidylinositol 3-kinase, and mitogen-activated protein kinase signaling pathways (Sigala et al., 2013). When subjected to an antioxidant-deficient diet, dopamine-β-hydroxylase knockout mice lacking NE exhibited significantly decreased proliferative activity of human primary HSCs and accumulation of TGF-β and hepatic α-smooth muscle actin, in contrast to control mice that demonstrated pronounced fibrosis (Oben et al., 2004).

Several studies have indicated that the elimination of hepatic sympathetic nerve endings may lead to the reversal of obesity-induced fatty liver (Hurr et al., 2019). These suggested that a comprehensive examination of the involvement of sympathetic nerves in the development, deterioration, and reversal of NASH holds promise for novel therapeutic approaches.

## 3 Animal models of NASH

Animal models are critical for studying disease mechanisms and evaluation of drug efficacy. Because the disease’s pathophysiology is uncertain, numerous models for replicating NASH exist; the following animal models have the highest reproducibility.

### 3.1 NASH models induced by a methionine–choline-deficient (MCD) diet

The MCD diet model has the advantages of simplicity and short time consumption. Hepatic steatosis, oxidative stress, hepatocyte mortality, and changes in cytokines and adipocytokines result from choline deficiency. After 2 weeks of feeding, MCD-fed mice developed extensive hepatic inflammation, followed by significant fibrosis within 6 weeks (Yamada et al., 2016). LSECs homeostasis was disrupted, and their specific gene profiles were altered. In an eNOS-dependent manner, Notch activation triggers LSECs maladaptation and worsens the NASH phenotype (Fang et al., 2022). Compared with mice fed a high-fat diet (HFD), the MCD diet induces inflammation, fibrosis, and liver cell apoptosis to develop faster and more severely and can better simulate the pathological results of severe human NASH. However, the MCD-induced animal model also has significant drawbacks, including significant weight loss, less pronounced ballooning of hepatocytes, and Mallory–Denk body deficiency, as well as lower levels of serum leptin, TG, fasting blood glucose, and insulin. In addition, various mice strains’ responses to the MCD diet varied significantly (Lau et al., 2017).

### 3.2 NASH model induced by choline-deficient L-amino-defined diet (CDAA)

CDAA diet substitutes an analogous combination of L-amino acids for proteins. The CDAA diet caused balloon denaturalization, inflammation, and peri-sinusoidal fibrosis, all of which are characteristics of human NASH (Ishioka et al., 2017). The livers of CDAA-fed mice showed significant lipid accumulation, hepatocellular inflammation, hepatocellular necrosis, and fibrosis. The increase in hepatic steatosis is related to the increased expression of adipogenic genes (*Dgat1* and *Srebp1c*) (Yang L. et al., 2014). Although the CDAA prevents weight loss, the mechanism of NASH may not be fully reflected in hepatic steatosis caused by VLDL-TG export deficiencies (Ipsen et al., 2020). Drug interventions can be conducted with this model, including those targeting NASH-related fibrosis.

### 3.3 Rifampicin-induced mouse NASH

Rifampicin increases CD36 gene expression, which is involved in hepatocyte FA absorption and FA synthesis. By activating the pregnane X receptor in the liver and causing the production of perilipin-2, the peroxisome proliferator-activated receptor (PPAR), and its downstream proteins, rifampicin increases fat infiltration into the liver and causes NASH (Ezhilarasan, 2023). CD-1 mice were given rifampicin (200 mg/kg) by gavage daily. One week later, the liver lipids significantly increased. FA synthesis, acetyl CoA carboxylase, and stearoyl-CoA-desaturase 1, three key genes for producing FA synthesis, all had higher mRNA levels (Huang et al., 2016). In mice, rifampicin 300 mg/kg given over 7 days significantly increased serum aminotransferase levels, total bilirubin and total bile acid levels, increased TG and total cholesterol content, cholesterol-activated 7α-hydroxylase, pregnane X receptor, FA synthesis, and Farnesol X receptor (FXR) mRNA expression, and pathology suggests that rifampicin-induced severe steatohepatitis and hepatocellular necrosis (Tan et al., 2020). This model is applicable to NASH caused by drug-induced liver injury.

### 3.4 NASH models induced by complex factors

NASH is a multifactorial disease. So the animal models are mostly built using composite factors, with two main categories: transgenic combined with dietary induction to resemble the actual possible causative factors and hepatic injurious agents combined with dietary induction.

### 3.5 NASH induced by tetracycline combined with an HFD in mice

By upregulating the enzymes fatty acid transferase (FAT or CD36) and diacylglycerol acyltransferase 2, tetracycline causes fatty acid transport and TG esterification (Choi et al., 2015). Intraperitoneal injection of tetracycline 40 mg/kg combined with a high-fat emulsion containing propylthiouracil and sodium deoxycholate at 10 mL/kg by gavage induced NASH in a much shorter period. It significantly elevates serum alanine aminotransferase, aspartate transaminase, and hepatic total cholesterol, and hepatocellular necrosis and intracellular vacuole formation of the liver could be seen in pathology by 7 days of continuous administration (Wanying et al., 2023).

### 3.6 NASH induced by an HFD in transgenic mice

Diet-induced NASH in mice: 129S1/SvImJ mice are used in the diet-induced NASH model. This isogenic line (B6/129) displays obesity, insulin resistance (IR), and hepatic steatosis. When fed a diet high in fat, sucrose, cholesterol, and sugar water for 16–24 weeks, both portal and central fibrosis are developed (F2). By week 52, most animals showed bridging fibrosis. In addition, this NASH mice model shows enhanced adipogenesis, endoplasmic reticulum stress, and apoptosis with a transcriptome similar to human NASH (Ipsen et al., 2020). In spite of the relatively long modeling time, this model is promising for studies of disease mechanisms and intervention strategies.

Foz/foz mice fed an HFD: The foz/foz mice carry a mutated Alms1 gene that encodes a protein in the primary ciliary matrix. Extremely obese and bulimic foz/foz mice also exhibit low IR adiponectin levels, elevated cholesterol, and liver steatosis. When given an HFD, foz/foz BALB/c and C57BL6/J mice acquired similar amounts of body weight, but the hepatic lesions in foz/foz C57BL6/J mice were more severe than those of the foz/foz BALB/c mice (Lau et al., 2017). Using this model, possible therapeutic interventions have been studied for NASH mice with obesity.

db/db mice fed an iron supplement or an MCD diet: NASH and fibrosis occur in mice with deficient leptin signaling as a result of iron overload. Hepatocyte expansion, fibrosis, elevated hepatic oxidative stress, activation of the inflammasome and hepatic inflammatory immune cells, and impaired hepatic mitochondrial fatty acid β-oxidation were observed in db/db mice fed high-iron diets as opposed to db/db mice fed normal chow. When given MCD chow, db/db mice developed severe hepatic fibrosis and inflammation (Lau et al., 2017).

KK-ay mice fed a CDAA-HFD: KK-ay mice with heterozygous Agouti mutations develop hyperphagia, obesity, hyperglycemia, IR, hepatic steatosis, and mild lobular inflammation. For ≤30 weeks, a CDAA-HFD or a Western diet was given to KK-ay mice to prevent weight loss. The liver inflammation and fibrosis of CDAA-HFD were more pronounced than in Western diets, but there was no significant difference compared to KK-ay and wild-type mice fed a CDAA-HFD. Conversely, the CDAA-HFD reduced insulin and glucose concentrations, contrary to typical phenotypes (Ipsen et al., 2020).

Melanocortin 4 receptor-deficient (Mc4r−/−) mice fed an HFD diet: Mc4r−/− fed an HFD diet for 20 weeks develop NASH with steatosis, ballooning degeneration, inflammation, and atrophic fatty liver (Ipsen et al., 2020). In addition to liver histological features, Mc4r−/− mice can exhibit IR and adipose tissue inflammation associated with obesity and develop well-differentiated HCC after feeding an HFD for 1 year (Itoh et al., 2011). This NASH model can be used to study dietary-induced liver steatosis, liver fibrosis, and HCC.

Gubra-Amylin NASH (GAN) diet-induced obesity (DIO): The GAN-DIO-NASH mouse model has shown good clinical translation to the histological, transcriptomic, and metabolic components of human illnesses. For 38–44 weeks, C57BL/6J mice were given a GAN diet high in saturated fat (40%) and cholesterol (2%), as well as fructose (22%), sucrose (10%), and sucrose. Compared to NASH patients, similarly morphologically characterized liver lesions were seen in this model. Glucose intolerance and obesity in GAN-DIO-NASH mice are associated with significant accumulation of liver lipid, hepatitis, and collagen deposition (Hansen et al., 2020). The GAN-DIO-NASH mouse model captures the different histological stages of NASH.

High-fat/cholesterol/cholate (HFCC) diet-induced NASH-associated fibrosis mice model: An HFCC diet for 9 weeks resulted in the development of NASH in two mice strains, C57BL/6J (sensitive to obesity) and A/J (relatively resistant to obesity). The A/J mice had more severe hepatic fibrosis and inflammation. The hepatic parenchyma of HFCC-A/J mice showed characteristic CD204-positive macrophage aggregation. Matrix-assisted laser desorption/ionization mass spectrometry imaging analysis demonstrated the presence of specific phospholipids in macrophages associated with fibrosis (Ichimura-Shimizu et al., 2022). HFCC-A/J mice provide fresh insights into how NASH-related fibrosis is triggered.

The “humanized” NASH model: As a humanized chimeric mouse model, human hepatocyte transplantation into fumarate acetate hydrolase-deficient mice is an important tool for researching drug metabolism, excretion, and toxicity in human-relevant systems. These mice develop NAFLD after being fed an HFD, exhibiting macrovesicular hepatocellular steatotic alterations and increased TG and cholesterol levels in the liver and serum. Humanized mice exposed to an HFD had inflammatory leukocyte infiltration in the liver, including macrophages, neutrophils, enlarged hepatocytes, activation of stellate cells, and the deposition of collagen. Humanized mice fed an HFD developed NASH phenotypes similar to human disease. RNA-seq analysis showed that numerous significant signaling pathways controlling the stability of the hepatic internal environment were damaged (Ma et al., 2022).

A short cycle, low cost, and the ability to reflect the key fundamental traits are all qualities that excellent animal models should possess. It is very difficult to set up a perfect NASH model using dexamethasone, tetracycline, or estrogen alone, for most of the liver tissues remain in a state of nonalcoholic simple fatty liver (Zhong et al., 2020; Quinn et al., 2018). Although feeding an MCD accurately reflects the features of NASH, it ignores the significant roles of obesity and IR in NASH. Selecting obese animals and feeding an MCD may be a better option for a NASH model.

## 4 Implementation status of related drug research and development strategies

### 4.1 Innovative drugs targeting the hepatic sinusoidal microenvironment

**FIGURE 2:**
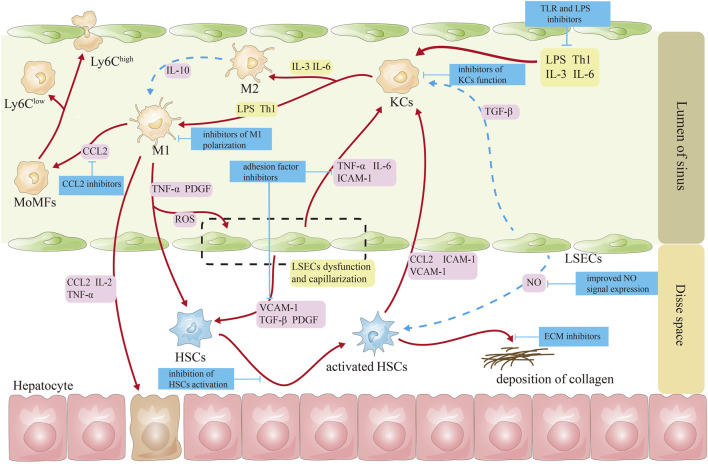
Schematic diagram of targeted treatment targets for cells in the hepatic sinusoids. Note (Eslam et al., 2020): The red line represents the activation effect (Rinella et al., 2023); the blue lines represent inhibitory effects (Younossi et al., 2023). CCL2, CC-chemokine ligand 2; HSCs, hepatic stellate cells; ICAM-1, intercellular adhesion molecule 1; IL-2, interleukin-2; IL-3, interleukin-3; IL-6, interleukin-6; KCs, Kupffer cells; LPS, lipopolysaccharide; LSECs, liver sinusoidal endothelial cells; MoMFs, monocyte-derived macrophages; NO, nitric oxide; PDGF, platelet-derived growth factor; ROS, reactive oxygen species; TGF-β, transforming growth factor β; Th1, helper T cell 1; TNF-α, tumor necrosis factor α; VCAM-1, vascular cell adhesion molecule 1.

The targeted therapeutic targets for the hepatic sinusoidal microenvironment are shown in Figure 2.

#### 4.1.1 Targeting macrophages

Regulating macrophage polarization: PPAR-γ is highly expressed in macrophages; activating PPAR-γ receptors can reduce the production macrophages of TNF-α and IL-1β by macrophages by inhibiting the nuclear factor κ-B signaling pathway and transform macrophages from M1 to M2 (Luo et al., 2017). Currently, saroglitazar, the PPAR-α/γ agonist, has been approved for sale in India and Mexico (Jain et al., 2018; Gawrieh et al., 2021). Aramchol reduces the fibrotic effect of HSCs by inhibiting HSCs stearoyl-CoA-desaturase 1 and activating PPAR-γ (Bhattacharya et al., 2021). An ongoing phase III clinical study is evaluating aramchol as a promising medication for NASH and fibrosis (Ratziu et al., 2021).

Pioglitazone is used widely as a hypoglycemic agent in treating type II diabetes and is currently in phase II clinical studies. It significantly reduces steatosis, lobular inflammation, and NAFLD activity scores, but it can also cause weight gain, fluid retention, bone loss, and increased fracture risk, which is more pronounced in older women (Cusi et al., 2016; Tahara, 2021). Pioglitazone treatment suppressed the activity of M1 macrophages and concurrently upregulated the expression of M2 macrophages in PPAR-γ-deficient macrophage mice (Gopalakrishnan et al., 2022). Pioglitazone decreased left ventricular remodeling in a rat model of myocardial infarction by altering macrophage polarization toward M2 macrophages (Konegawa et al., 2022). Pioglitazone suppressed the polarization of M1 macrophages and enhanced the polarization of M2 macrophages through the PPAR-γ-miR-23-Irf1/Pknox1 pathway, leading to a reduction in inflammatory tissue damage (Chen et al., 2019). Galectin-3, mainly expressed in macrophages, participates in various chronic liver disease-induced liver fibrosis by promoting macrophage differentiation and secreting chemokines. Galectin-3 promotes concanavalin A-induced hepatitis and exacerbates liver injury by enhancing the activation of T-lymphocytes, natural killer T cells, and dendritic cells, promoting cytokine production, inhibiting the polarization of M2 macrophages, and preventing the death of monocytes (Radosavljevic et al., 2012). As a candidate new drug for preventing and treating NASH, in phase I clinical trials, the galectin-3 inhibitor, belapectin, was found to be safe and well tolerated, and it is currently undergoing phase III clinical studies (Chalasani et al., 2020; Harrison et al., 2016).

Inhibition of MoMF recruitment in the liver: MoMF recruitment depends on several chemokines activated and secreted by macrophages and activated HSCs to promote liver fibrosis. Cenicriviroc, a combined antagonist of CC-chemokine receptor 2/5, decreases pro-inflammatory monocyte, macrophage, and hepatic stellate cell recruitment, migration, infiltration, and activation. However, its antifibrotic actions are circumscribed (Ratziu et al., 2020).

#### 4.1.2 Targeting hepatic sinusoidal endothelial cells

Affecting the expression of NO signals on LSECs: In addition to maintaining LSECs morphology and function, NO regulates hepatic lipid and glucose homeostasis (Maslak et al., 2015). During NAFLD, impaired insulin signaling, decreased protein kinase B (AKT)-dependent eNOS phosphorylation, and reduced NO synthesis and release ultimately lead to increased hepatic vascular resistance. Therefore, insulin sensitizers can improve LSECs function by mediating the NO pathway, thereby alleviating NASH (McConnell et al., 2023). Glucagon-like peptide-1 and its receptor agonists—liraglutide: *In vitro* and *in vivo* studies have shown that liraglutide improves vascular endothelial cell function by inhibiting the induction of fibrinogen activator inhibitor 1 and vascular adhesion molecule expression in human vascular endothelial cells while increasing NO synthase activity (Del Olmo-Garcia and Merino-Torres, 2018). In addition, liraglutide improves hindlimb ischemia in type II diabetic mice by increasing angiogenesis *in vitro* and *in vivo via* activation of AKT/eNOS and phospho-extracellular signaling-associated kinase 1 and 2 signaling (Zhu et al., 2023). Liraglutide demonstrated better pharmacodynamic effects in NASH animals, including weight loss, decreased liver lipid content, and improved liver NASH pathological score (Armstrong et al., 2016). Pan-caspase inhibitor emricasan can increase the fenestration of LSECs, reduce the expression of Sinus von Willebrand factor, and increase eNOS expression and the content of NO and cyclic guanosine monophosphate, which decreases hepatic vein pressure (Gracia-Sancho et al., 2019). Unfortunately, it has not received encouraging news in improving fibrosis in NASH patients (Harrison et al., 2020).

Inhibition of cell adhesion on the surface of LSECs: Extracellular vesicles derived from lipotoxic hepatocytes are rich in active integrin β1. Integrin β1 mediates the adhesion of monocytes to LSECs. Integrin β1 antibody can significantly improve liver inflammation in NASH mice (Guo et al., 2019).

#### 4.1.3 Targeting HSCs

HSCs express several nuclear receptors, including FXR and thyroid hormone receptor-α, which regulate the expression of pro-inflammatory and fibrogenic cytokines in the liver and promote the transformation of HSCs into fibroblasts (Königshofer et al., 2021). FXR is involved in energy expenditure and metabolism. Several FXR agonists, including tropifexor and EDP-305, are currently in phase II clinical study (Sanyal et al., 2023a; Ratziu et al., 2022; Ratziu et al., 2023). The FXR agonist obeticholic acid (OCA) arouses the greatest attention and exhibits promising results. OCA inhibits the activation of hematopoietic stem cells by regulating bile acid levels (Zhou et al., 2019). OCA is believed to be the first NASH drug to be approved for marketing. In a phase II trial (FLINT), OCA significantly improved inflammation and fibrosis in NASH patients (Neuschwander-Tetri et al., 2015). This serious illness may soon receive its first drug approval after positive phase III clinical trial results. OCA significantly improves fibrosis and is well tolerated after long-term administration (Sanyal et al., 2023b). So far, OCA has achieved a phased victory in treating liver diseases.

Inhibiting HSCs activation: the fibroblast growth factor 21 analog, efruxifermin, can directly inhibit the differentiation of HSCs into myofibroblasts and simultaneously inhibit the activation of KCs, improving liver fibrosis in NASH patients (Tillman and Rolph, 2020). Although it is still in phase III clinical trials, aldafermin, a fibroblast growth factor 19 analog, can inhibit bile acid synthesis, regulate metabolic homeostasis, promote lipolysis, and clinically reduce hepatic lipids content in patients with NASH (Harrison et al., 2022).

Promoting apoptosis of HSCs: Apoptotic signal-regulated kinase 1 (ASK1) is activated under oxidative stress, resulting in worsening liver inflammation, apoptosis, and fibrosis (Budas et al., 2016). Selonsertib, an inhibitor of ASK1, can block the ASK1/MAPK pathway. As a result, liver fibrosis may improve when HSCs are inhibited in proliferation and ECM components, and autophagy and apoptosis are induced through the c-Jun N-terminal kinase and AKT signaling pathways (Yoon et al., 2020). Phase III clinical trials for this drug are currently underway.

## 5 Future perspective

KCs, HSCs, LSECs, and hepatocytes are formed entirely by the nervous and humoral systems. They work independently, and their interplay contributes to the homeostasis of hepatic sinusoids in this condition. Once the balance is broken, liver injury, such as NASH, will happen. Currently, new drugs aimed at KCs, HSCs, and LSECs are under development, but drugs targeting the nervous system are not yet found in the literature. The underlying cause may be attributed to the insufficient specificity of receptors or neurotransmitters within the hepatic nervous system, which complicates the identification of compounds exhibiting low toxicity and high efficacy that selectively target the hepatic nervous system. In this context, employing pharmacological strategies to selectively inhibit the release or depletion of hepatic neurotransmitters could be considered as a means to expedite the restoration of hepatic sinusoidal homeostasis and effectively reverse NASH.

Due to the multifaceted nature of the occurrence and progression of NASH, therapeutic drug development has predominantly concentrated on a singular target, resulting in the absence of optimal treatment. Our research illustrates that natural remedies have the potential to effectively prevent and manage NASH by modulating cholesterol and TG metabolism, inhibiting the P38 MAPK pathway to mitigate inflammation, and decreasing TGF-β1 expression to attenuate liver fibrosis (Yang Q. et al., 2014; Guo X. et al., 2022; Yingyang et al., 2014; Xiaoping et al., 2021).

So far, dozens of candidate compounds have been discovered for NASH, but there is a significant divergence between preclinical and clinical efficacy. First, there are species differences between animals and humans, which are not only related to the great differences in diet and living habits but also to the significant differences in the structure of the liver. Second, the current NASH animal model has obvious shortcomings; even the classical MCD mice cannot be used to investigate the role of liver lipid accumulation caused by IR in the occurrence and development of NASH. Third, because the specific target of NASH has not yet been found, comprehensive therapy has shown unexpected curative effects in the prognosis of NASH; therefore, many companies are accelerating the development of compound drugs (traditional Chinese medicine or Western medicine) to conquer NASH, but to date, no exciting news has appeared.

The insufficient systematic elucidation of the pathogenesis of NASH and the absence of optimal therapeutic agents can be attributed primarily to the tendency of researchers to concentrate on one single target or cell type. This approach overlooks the integrative nature of the cellular interactions within the hepatic sinusoids. Cells exhibit intricate interactions, and dysfunction in one domain will invariably influence the entire system. Consequently, future research in pathogenesis and novel drug development must adopt a holistic approach to effectively analyze and address these challenges. Nevertheless, owing to methodological constraints in the investigation of hepatic sinusoidal structural units, contemporary research predominantly relies on animal models, resulting in a paucity of clinical research data and rendering the identification of characteristic indicators for clinical diagnosis unfeasible.
